# ERK Positive Feedback Regulates a Widespread Network of Tyrosine Phosphorylation Sites across Canonical T Cell Signaling and Actin Cytoskeletal Proteins in Jurkat T Cells

**DOI:** 10.1371/journal.pone.0069641

**Published:** 2013-07-17

**Authors:** Ynes A. Helou, Vinh Nguyen, Samantha P. Beik, Arthur R. Salomon

**Affiliations:** 1 Department of Molecular Pharmacology, Physiology, and Biotechnology, Brown University, Providence, Rhode Island, United States of America; 2 Department of Molecular Biology, Cell Biology, and Biochemistry, Brown University, Providence, Rhode Island, United States of America; 3 Department of Chemistry, Brown University, Providence, Rhode Island, United States of America; University of Torino, Italy

## Abstract

Competing positive and negative signaling feedback pathways play a critical role in tuning the sensitivity of T cell receptor activation by creating an ultrasensitive, bistable switch to selectively enhance responses to foreign ligands while suppressing signals from self peptides. In response to T cell receptor agonist engagement, ERK is activated to positively regulate T cell receptor signaling through phosphorylation of Ser^59^ Lck. To obtain a wide-scale view of the role of ERK in propagating T cell receptor signaling, a quantitative phosphoproteomic analysis of 322 tyrosine phosphorylation sites by mass spectrometry was performed on the human Jurkat T cell line in the presence of U0126, an inhibitor of ERK activation. Relative to controls, U0126-treated cells showed constitutive decreases in phosphorylation through a T cell receptor stimulation time course on tyrosine residues found on upstream signaling proteins (CD3 chains, Lck, ZAP-70), as well as downstream signaling proteins (VAV1, PLCγ1, Itk, NCK1). Additional constitutive decreases in phosphorylation were found on the majority of identified proteins implicated in the regulation of actin cytoskeleton pathway. Although the majority of identified sites on T cell receptor signaling proteins showed decreases in phosphorylation, Tyr^598^ of ZAP-70 showed elevated phosphorylation in response to U0126 treatment, suggesting differential regulation of this site via ERK feedback. These findings shed new light on ERK’s role in positive feedback in T cell receptor signaling and reveal novel signaling events that are regulated by this kinase, which may fine tune T cell receptor activation.

## Introduction

The adaptive immune response relies the T cell receptor (TCR) to discriminate between foreign and self antigen. In canonical T cell activation, signaling events induced by the interaction between a TCR and peptide-major histocompatibility complex (MHC) agonist generates a set of cellular physiological changes that culminate in T cell proliferation, differentiation, and cytokine secretion. Upon activation of the TCR, the Src family protein tyrosine kinases Lck and Fyn phosphorylate the TCR CD3 chain immunoreceptor tyrosine-based activation motifs (ITAMs). Once fully phosphorylated, the ITAMs serve as binding sites for the Syk family protein tyrosine kinase ζ-chain associated protein of 70 kDa (ZAP-70), which is recruited to the TCR. There, ZAP-70 is phosphorylated and activated by the Src kinase Lck. A number of signaling proteins, including the scaffolding proteins linker for activation of T cells (LAT) and SH2 domain-containing leukocyte protein of 76kDa (SLP-76) are subsequently phosphorylated by active ZAP-70. Once phosphorylated, LAT and SLP-76 form a signalosome complex essential for the assembly and activation of downstream signaling proteins. [1]–[3].

**Figure 1:**
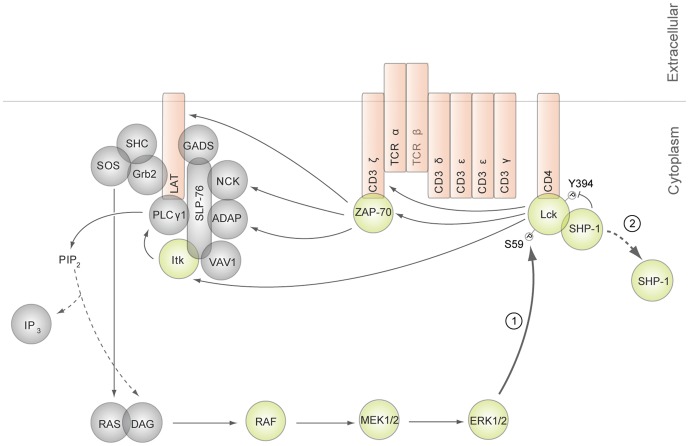
ERK positive feedback model. (1) ERK phosphorylation at Ser^59^ Lck leads to modification of Lck’s SH2 domain. (2) Modification of Lck’s SH2 domain prevents SHP-1 association with Lck.

Proper T cell discrimination between structurally similar self and foreign antigens is complicated by the continuous signal inputs to the TCR signaling machinery from a plethora of low affinity self antigens. Competing positive and negative feedback pathways constitute one of the central mechanisms utilized to tune the sensitivity of TCR activation to self and foreign ligands [1], [4], [5]. Downstream of the TCR, numerous proteins involved in feedback pathways that regulate TCR activation have been characterized. Proteins reported to function in negative feedback mechanisms in TCR signaling include C-terminal Src kinase (Csk), Dok-1, Dok-2, and CBL [6]–[9]. One particular negative feedback pathway that occurs upon engagement of the TCR by a weak agonist or antagonist is mediated by SH2-containing protein tyrosine phosphatase 1 (SHP-1). This pathway is initiated by Lck-dependent phosphorylation and activation of SHP-1. Active SHP-1 then mediates inactivation of Lck via dephosphorylation of its active site, Tyr^394^, resulting in reduced phosphorylation of the CD3 ζ chains, and attenuation of intracellular signaling by the TCR [4]. Positive feedback mechanisms that promote T cell activation have also been observed in T cells, but are less defined [4], [5], [10], [11]. In particular, it has been reported that in response to TCR interaction with high affinity agonists, ERK is activated to positively regulate TCR signaling through Lck (Figure 1) [4], [12]. Upon TCR agonist engagement, Lck becomes phosphorylated at Ser^59^ by ERK [13], [14] leading to the modification of Lck’s Src homology 2 (SH2) domain, and consequently, a reduction in the accessibility or affinity for phosphoproteins to bind [15]. Specifically, modification of the SH2 domain of Lck interferes with the recruitment of phosphorylated SHP-1, preventing Lck inactivation and allowing for a longer-lasting TCR-induced stimulatory signal [4].

The current understanding of ERK’s role in TCR positive feedback signaling has been limited to immunoblot analysis of select TCR proximal proteins. Because of the critical role of Lck in initiation of the TCR signaling cascade, we hypothesize that the loss of ERK-mediated phosphorylation of Ser^59^ Lck will result in SHP-1-mediated dephosphorylation of its activation site, and consequently, global decreases in phosphorylation on the majority of TCR proximal signaling proteins. To elucidate the function of this kinase in regulating the TCR signaling pathway, a wide-scale view of temporal changes in the tyrosine phosphorylation of TCR signaling components is required. Quantitative mass spectrometry-based phosphoproteomics is a powerful means to achieve this goal by enabling the wide-scale identification of sites on proteins phosphorylated in T cells, as well as the quantification of protein phosphorylation dynamics, as demonstrated in various studies [16]–[21]. Here, a quantitative mass spectrometry-based phosphoproteomics approach was implemented to investigate the impact of ERK feedback on TCR signaling.

## Materials and Methods

### Inhibitor Treatment

Jurkat T cells (E6-1 clone) were obtained from American Tissue Culture Collection (Manassas, VA). To determine the optimal inhibitor concentration for inhibition of ERK activation, Jurkat T cells were treated with U0126 (Cell Signaling Technology, Danvers, MA) at various concentrations (0 µM, 10 µM or 20 µM) at 37°C. Inhibition of MEK1/2 was quantified using Western blots. In phosphoproteomic experiments, prior to stimulation, Jurkat T cells were treated with 20 µM of U0126 for 2.5 hours at 37°C.

### Cell Culture, SILAC Labeling, and T Cell Stimulation

Jurkat cells were initially maintained in RPMI 1640 medium (HyClone, Logan, UT) supplemented with 10% heat-inactivated undialyzed FBS (Invitrogen, Carlsbad, CA), 2 mM L-glutamine, 100 U/ml penicillin G, and 100 µg/ml streptomycin (HyClone) in a humidified incubator with 5% CO_2_ at 37°C. Stable isotope labeling with amino acids in cell culture (SILAC) was performed as described [20]. Briefly, cells growing in logarithmic phase were washed twice with SILAC RPMI 1640 medium (Pierce Biotechnology, Rockford, IL) without arginine and lysine and reconstituted in SILAC RPMI 1640 medium containing either ^12^C_6_, ^14^N_4_ arginine and ^12^C_6_, ^14^N_2_ lysine (Sigma, St. Louis, MO) or ^13^C_6_, ^15^N_4_ arginine and ^13^C_6_, ^15^N_2_ lysine (Cambridge Isotope Laboratories, Andover, MA) supplemented with 10% heat-inactivated dialyzed FBS (Invitrogen), 2 mM L-glutamine, 100 U/ml penicillin G, 100 µg/ml streptomycin in a humidified incubator with 5% CO_2_ at 37°C for 7 cell doublings. The concentration of lysine and arginine used in SILAC labeling of Jurkat cells in experiments described here was 0.22 mM and 0.38 mM, respectively.

Prior to TCR stimulation, Jurkat cells cultured in heavy SILAC medium were treated with 20 µM U0126 for 2.5 hours, and cells cultured in light SILAC medium were treated with a vehicle control (0.1% DMSO). To stimulate the TCR, cells were reconstituted at a concentration of 1×10^8^ cells/ml in PBS. For each stimulation time point, 1×10^8^ cells were treated with OKT3 and OKT4 antibodies (eBioscience, San Diego, CA) at a concentration of 2.5 µg/ml of each antibody for 30 seconds at 37°C. Cells were then cross-linked with 22 µg/ml of goat anti-mouse IgG (Jackson ImmunoResearch, West Grove, PA) and incubated at 37°C for 0, 2.5, 5, or 10 minutes. In total, 4 biological replicates of each time course stimulation were prepared for each condition.

### Cell Lysis, Protein Reduction, Alkylation, Digestion, and Peptide Immunoprecipitation

To halt the stimulation, cells were placed in lysis buffer (9 M urea, 1 mM sodium orthovanadate, 20 mM HEPES, 2.5 mM sodium pyrophosphate, 1 mM β-glycerophosphate, pH 8.0) and incubated for 20 minutes at 4°C. Lysates were then sonicated at a 30 watt output with 2 bursts of 30 seconds each and cleared at 20,000×g for 15 minutes at 4°C. Protein concentrations were measured by the DC Protein Assay (Bio-Rad, Hercules, CA). Once protein concentrations were determined, cell lysates from inhibitor-treated and DMSO-treated cells were combined in a 1∶1 protein concentration ratio, reduced with 45 mM DTT for 20 minutes at 60°C, and alkylated with 100 mM iodoacetamide for 15 minutes at room temperature (RT) in the dark. Cell lysates were then diluted 4-fold with 20 mM HEPES buffer, pH 8.0 and digested with TPCK-treated trypsin (Worthington, Lakewood, NJ) in a 1∶1 (w/w) trypsin:protein ratio overnight at RT. Tryptic peptides were acidified to pH 2.0 with 20% trifluoroacetic acid (TFA), cleared at 1,800×g for 5 minutes at RT, and desalted using C18 Sep-Pak plus cartridges (Waters, Milford, MA) as described [20], with the exception that TFA was used instead of acetic acid. Eluents containing peptides were lyophilized for 48 hours to dryness.

Peptide immunoprecipitation was performed using p-Tyr-100 phosphotyrosine antibody beads (Cell Signaling Technology). Lyophilized peptides from each time point were reconstituted in ice-cold immunoaffinity purification (IAP) buffer (50 mM MOPS pH 7.2, 10 mM sodium phosphate, 50 mM NaCl) and dissolved through gentle shaking for 30 minutes at RT and brief sonication in a sonicator water bath. Prior to peptide immunoprecipitation, a 5 pmol fraction of synthetic phosphopeptide LIEDAEpYTAK was added to each time point sample as an exogenous quantitation standard. Peptide solutions were then cleared at 1,800×g for 5 minutes at RT, combined with p-Tyr-100 phosphotyrosine antibody beads, and incubated for 2 hours at 4°C. Beads were washed 3 times with IAP buffer and twice with cold Milli-Q water, and eluted with 0.15% TFA. Samples were then desalted using Zip-Tip C18 columns (EMD Millipore, Billerica, MA).

### Western Blotting

Total cellular protein from 9 M urea cell lysates was diluted in sample loading buffer (4% SDS, 125 mM Tris-HCl, pH 6.8, 20% v/v glycerol, 5% 2-mercaptoethanol, 0.01% bromophenol blue) for each proteomic sample. Equal amounts of protein, as measured by the DC Protein Assay, were separated by 4–20% gradient SDS-PAGE on a Precise Tris-HEPES gel (Thermo Fisher Scientific, Waltham, MA), and electroblotted onto an Immobilon membrane (EMD Millipore). The membrane was blocked with Odyssey blocking buffer (Li-Cor, Lincoln, NE) and then incubated with primary antibody in blocking buffer. With the exception of anti-Lck (phospho-Ser^59^) (Assay Biotechnology, Sunnyvale, CA) and anti-phosphotyrosine antibody, clone 4G10 (EMD Millipore), the following primary antibodies were from Cell Signaling Technologies: anti-phospho-p44/p42 MAPK (ERK1/2) (Thr^202^/Tyr^204^), anti-p44/42 MAPK (ERK1/2), anti-phospho-ZAP-70 (Tyr^493^), anti-ZAP-70, anti-GAPDH, and anti-Lck. Bound antibodies were detected with anti-rabbit IgG and anti-mouse IgG directly conjugated to an IRDye (Li-Cor). Bands were visualized and quantified using the Odyssey CLx Imaging System (Li-Cor).

### Automated Nano-LC/MS

Tryptic peptides were analyzed by a fully automated phosphoproteomic technology platform [22], [23]. Phosphopeptides were eluted into a Linear Trap Quadropole (LTQ) Orbitrap Velos mass spectrometer (Thermo Fisher Scientific) through a PicoFrit analytical column (360 µm outer diameter 75 µm inner diameter-fused silica with 12 cm of 3-µm Monitor C18 particles; New Objective, Woburn, MA) with a reversed-phase gradient (0–70% 0.1 M acetic acid in acetonitrile in 90 minutes). Spectra were collected in positive ion mode and in cycles of one full MS scan in the Orbitrap (m/z 400–1800) followed by data-dependent tandem mass spectrometry (MS/MS) scans in the LTQ Velos, sequentially of the ten most abundant ions in each MS scan with charge state screening for +1, +2, +3 ions and dynamic exclusion time of 30 seconds. The automatic gain control was 1,000,000 for the Orbitrap scan and 10,000 for the LTQ scans. The maximum ion time was 100 milliseconds for the LTQ scan and 500 milliseconds for the Orbitrap full scan. Orbitrap resolution was set at 60,000.

### Database Analysis

MS/MS spectra were searched against the human UNIPROT non-redundant “complete proteome set” protein database using Mascot (Matrix Science) [24]. Peak lists were generated using extract_msn.exe (1/10/11) using a mass range of 600–4500. The UNIPROT human database contained 88,832 protein sequence entries. The Mascot database search was performed with the following parameters: trypsin enzyme specificity, 2 possible missed cleavages, 7 ppm mass tolerance for precursor ions, and 0.5 Da mass tolerance for fragment ions. Search parameters specified a differential modification of phosphorylation (+79.9663 Da) on serine, threonine, and tyrosine residues and methionine oxidation (+15.9949 Da) as well as a static modification of carbamidomethylation (+57.0215 Da) on cysteine. Search parameters also included a differential modification for arginine (+10.00827 Da) and lysine (+8.01420 Da) amino acids. To provide high confidence phosphopeptide sequence assignments, Mascot results were filtered by Mowse score (>10) and precursor mass error (<2 ppm).

After the database search, resulting peptide assignments were filtered down to 1% false discovery rate (FDR) by a logistic spectral score [25]. FDR was estimated with the decoy database approach after final assembly of non-redundant data into heatmaps [26]. To validate the position of the phosphorylation sites, the Ascore algorithm [27] was applied to all data, and the reported phosphorylation site position reflected the top Ascore prediction. Ascore probabilities are reported in the full data table (Dataset S1).

### Quantitation of Relative Phosphopeptide Abundance

Relative quantitation of phosphopeptide abundance was performed via calculation of select ion chromatogram (SIC) peak areas for heavy and light SILAC-labeled phosphopeptides identified from 4 biological replicate experiments. Peak areas were calculated by inspection of SICs using software programmed in Microsoft Visual Basic 6.0 based on Xcalibur Development kit 2.0 SR2 (Thermo Fisher Scientific). Quantitative data was calculated automatically for every assigned phosphopeptide using the ICIS algorithm available in the Xcalibur XDK.

For label-free comparison of phosphopeptide abundance in DMSO-treated control cells across the time course of TCR stimulation, individual time point SICs were normalized to an exogenously spiked standard phosphopeptide LIEDAEpYTAK peak area in the same time point. The LIEDAEpYTAK phosphopeptide was added at the same amount in each time point and replicate sample and accompanied cellular phosphopeptides through peptide immunoprecipitation and reversed-phase elution into the mass spectrometer. A label-free data heatmap was generated for comparison of phosphopeptides in DMSO-treated control cells through the time course of receptor stimulation, representing the average of 4 biological replicate experiments. The magnitude of change of the heatmap color was calculated from the natural log of the ratio of the fold change of each individual phosphopeptide peak area compared with the geometric mean for that phosphopeptide across all time points. In the heatmap representation, the geometric mean of a given phosphopeptide across all time points was set to the color black. A blue color represented below average abundance, while yellow represented above average abundance for each unique phosphopeptide. Blanks in the heatmap indicated that a clearly defined SIC peak was not observed for that phosphopeptide in any of the replicate analyses for that time point. The heatmap colors were generated from the average of the LIEDAEpYTAK standard phosphopeptide normalized SICs in the four replicate experiments. The coefficient of variation (CV) of the 4 biological replicates was calculated for each heatmap square. P values were calculated for each time point compared to the time point with the minimal average peak area for that phosphopeptide. A q value is defined as the measure of the minimum FDR at which a test can be called significant [28]. For each time point, q values for multiple hypothesis tests were calculated based on the determined p values using the R package QVALUE as previously described [29], [30].

In a second type of heatmap, SILAC ratios from the average of 4 biological replicate experiments corresponding to phosphopeptide abundance differences between inhibitor-treated and DMSO-treated control cells across the time course of receptor stimulation were represented. For the SILAC heatmaps, a black color represented a ratio of 1 between the two cell treatments for a given phosphopeptide at that time point. A red color represented less abundance, and green represented higher abundance of the given phosphopeptide in the inhibitor-treated cells compared with the DMSO-treated control cells. The magnitude of change of the heatmap color was calculated as described [20]. The CV was calculated for each SILAC ratio heatmap square across the four replicate analyses. The q values were also calculated for each SILAC heatmap square to assess the statistical significance of phosphopeptide abundance changes between U0126-treated and DMSO-treated measurements for each time point.

## Results and Discussion

**Figure 2:**
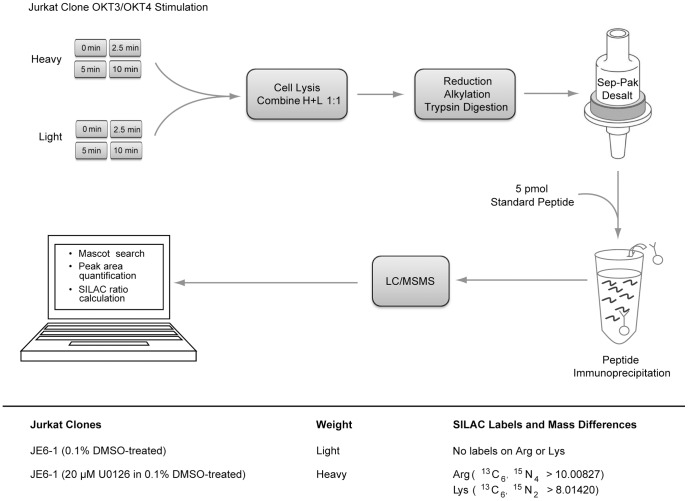
Experimental protocol. Human Jurkat T cells were incubated with light or heavy stable isotope-labeled arginine and lysine amino acids, physically differentiating the two proteomes by a shift in molecular weights. Cells were treated with either U0126 (heavy-labeled cells) or DMSO (light-labeled cells) for 2.5 hours prior to stimulation. Each cell population was then pre-incubated with OKT3 and OKT4 antibodies for 30 seconds at 37°C and then cross-linked with IgG at 37°C for the times indicated.

**Figure 3:**
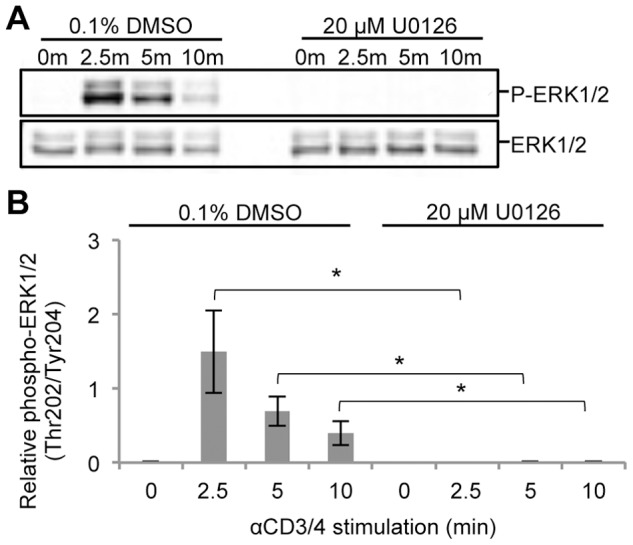
U0126 inhibits ERK1/2 activation across a time course of TCR stimulation. Jurkat T cells were treated with either 20 µM U0126 or 0.1% DMSO for 2.5 hours prior to TCR stimulation. (A) Cell lysates from a time course of TCR stimulation in the presence of 0.1% DMSO or 20 µM U0126 were separated by SDS-PAGE and immunodetected with phospho-p44/p42 MAPK (ERK1/2) and p44/42 MAPK (ERK1/2) specific antibodies. (B) Densitometric analysis of phospho-ERK1/2 levels normalized to ERK1/2 levels was performed. Shown is the mean ± S.D. from 4 biological replicate experiments. Statistically significant differences in relative phospho-ERK1/2 levels between U0126-treated and DMSO-treated cells for each time point are indicated with an asterisk (*-p value <0.01).

**Figure 4:**
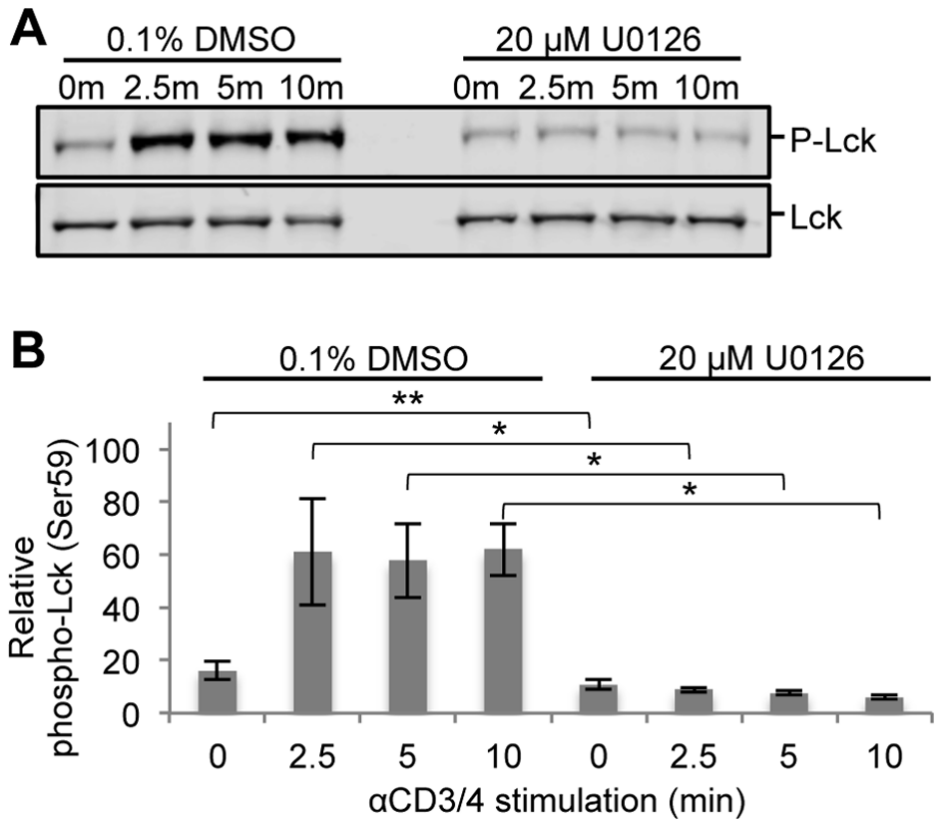
Phosphorylation of Ser^59^ Lck across a time course of TCR stimulation. (A) Cell lysates from a time course of TCR stimulation in the presence of 0.1% DMSO or 20 µM U0126 were separated by SDS-PAGE and immunodetected with phospho-Ser^59^ Lck and Lck specific antibodies. (B) Densitometric analysis of phospho-Ser^59^ Lck levels normalized to Lck levels was performed. Shown is the mean ± S.D. from 4 biological replicate experiments. Statistically significant differences in relative phospho-Ser^59^ levels between U0126-treated and DMSO-treated cells for each time point are indicated with an asterisk (*-p value <0.007, **-p value <0.015).

ERK has been reported to regulate a positive feedback mechanism essential to TCR sensitivity and selectivity towards foreign antigens [4]. Using the human Jurkat T cell leukemia cell line, the preferred model system for studying TCR signaling [31], a quantitative mass spectrometry-based phosphoproteomic strategy was undertaken to investigate the systems-wide effects of ERK feedback in TCR signaling (Figure 2). To inhibit ERK feedback, Jurkat T cells were treated with 20 µM U0126 for 2.5 hours prior to TCR stimulation, which led to complete inhibition of ERK phosphorylation at its active sites in all stimulated conditions relative to controls (Figure 3A, B, Figure S1). According to the literature, U0126 selectively inhibits ERK activation [16], [32], [33], and has been shown not to affect the JNK or p38 pathways in T cells [34]. Importantly, following inhibitor treatment of cells, ERK protein levels remained stable relative to DMSO treatment (Figure S2). To confirm ERK-mediated feedback on Lck, levels of phospho-Ser^59^ Lck were quantified by Western blot. Phosphorylation of Ser^59^ Lck increased in response to TCR stimulation in 0.1% DMSO treated cells. As expected, U0126-treated cells showed no increase in the phosphorylation of this site in response to TCR stimulation (Figure 4A, B).

### Phosphoproteomic Sample Quantitation and Statistical Analysis

SILAC-labeled cells were treated with U0126 or the vehicle control DMSO, and a receptor stimulation time course experiment of four time points was performed. In total, 4 biological replicates of each time course and treatment condition were prepared. After MS processing of phosphoproteomic samples and generation of raw MS phosphopeptide identifications, high quality sequence assignments were determined using stringent criteria as described in Materials and Methods. Relative quantitation of phosphopeptide abundance via calculation of SIC peak areas was performed for each light SILAC-labeled phosphopeptide (DMSO-treated) in each time point. SILAC ratios were also calculated by comparison of the SIC peak areas of phosphopeptides from inhibitor-treated cells to their DMSO-treated counterparts. For each sequenced phosphopeptide, two different visual representations of quantitative data in the form of heatmaps were generated to reflect either the label-free or SILAC ratio data.

To ascertain biologically relevant conclusions, stringent statistical tests were performed on the SIC peak areas of confidently assigned phosphopeptides that assessed the significance of quantification of phosphopeptide abundance across replicate observations. Q values assessed the significance of phosphopeptide abundance changes across the 4 time points of TCR stimulation in label-free heatmaps or between U0126-treated and DMSO-treated measurements for each time point in SILAC heatmaps. Statistical significance was only attributed to quantified phosphopeptides with q values less than 0.02. The reproducibility of SILAC analysis among replicate experiments was confirmed by evaluating the correlation of log2 transformed SILAC ratios between each biological replicate in scatter plots (Figure S3). A complete list of sequence and phosphorylation site assignments of all identified phosphopeptides with corresponding SIC peak areas, SILAC ratios, and statistics are reported in the full data table (Dataset S1).

**Figure 5:**
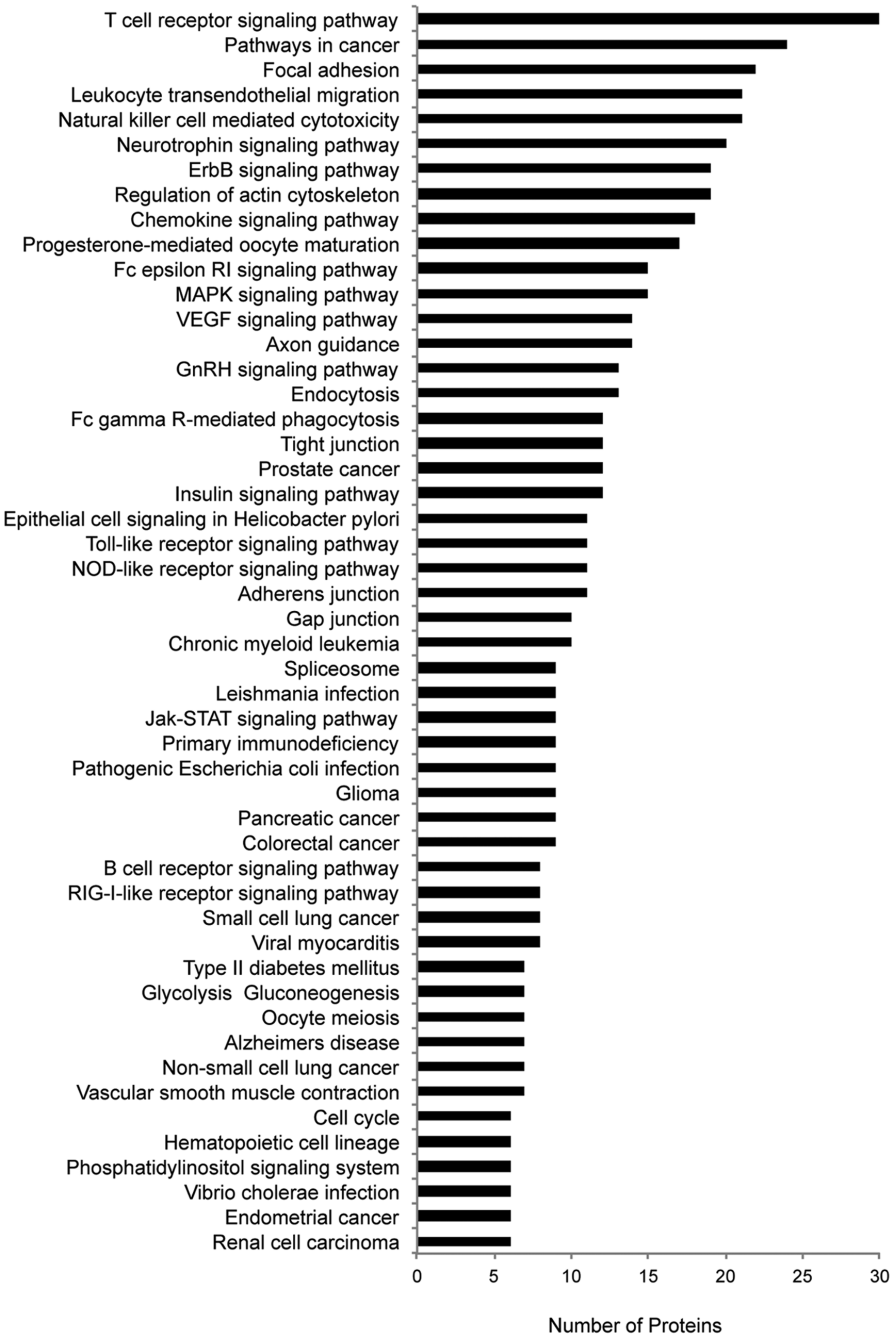
KEGG pathway analysis. A KEGG analysis of confidently identified phosphopeptides (1% FDR) from four biological replicate experiments was performed.

**Figure 6:**
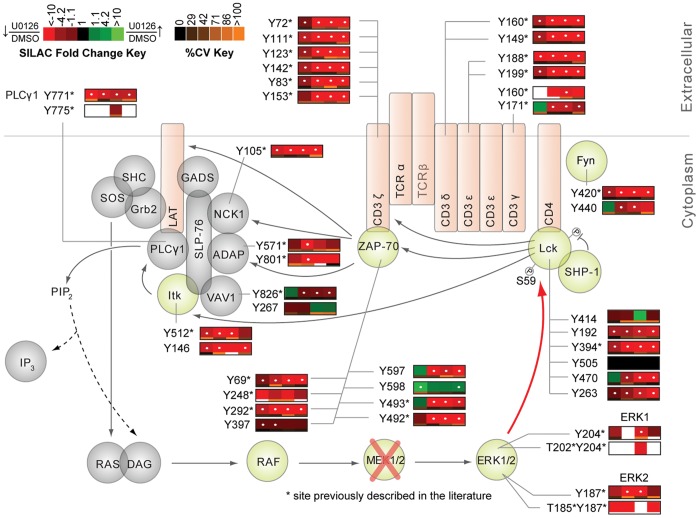
Effects of U0126 on the canonical TCR signaling pathway. Depicted is a model of ERK positive feedback with quantitative U0126-treated to DMSO-treated SILAC ratio heatmaps beside individual proteins, corresponding to the changes in phosphorylation between the two conditions across the four time points of TCR stimulation. Heatmaps were calculated from the averages of four biological replicate experiments. Green represents elevated phosphorylation in response to U0126 treatment relative to DMSO-treated controls, whereas red represents a decrease in phosphorylation relative to DMSO-treated controls. Blanks in the heatmap indicate that a clearly defined SIC peak was not observed for that phosphopeptide in that time point. Black represents no change. White dots within the heatmap indicate a statistically significant difference (q value <0.02) in the comparison between U0126-treated and control DMSO-treated SILAC ratios for that time point. Below each heatmap square is a color bar representing the percent CV for that time point. Orange represents a high degree of variation, while black represents a low degree of variation amongst the replicate analyses. Blanks indicate a lack of replicate data required to accurately determine the CV.

**Figure 7:**
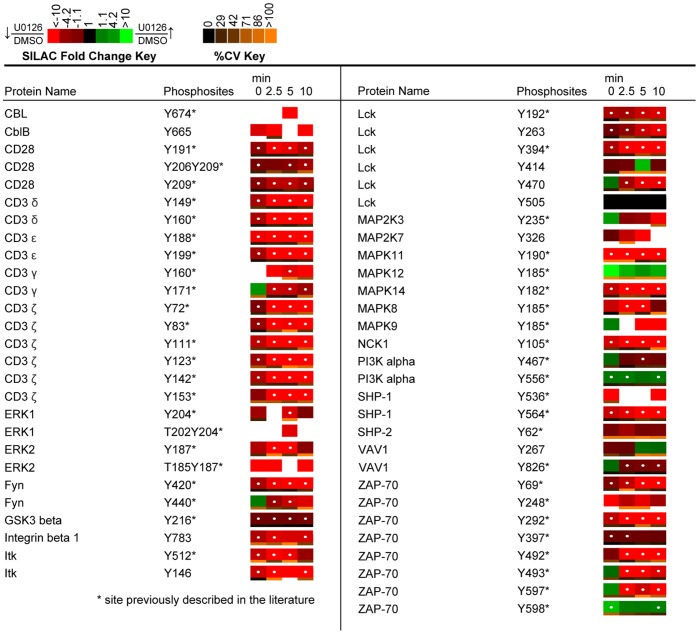
Quantitative phosphoproteomic analysis of proteins associated with the KEGG TCR signaling pathway. Depicted is a SILAC heatmap representation of temporal changes in tyrosine phosphorylation of proteins associated with the KEGG TCR signaling pathway. Heatmaps were calculated from the averages of four biological replicate experiments. SILAC ratios between U0126-treated and control DMSO-treated Jurkat T cells are represented for each phosphopeptide and time point. A white dot within the SILAC heatmaps indicate a statistically significant difference (q value <0.02) in the comparison between U0126-treated and control SILAC ratios for that time point. Abbreviations: GSK3 beta, Glycogen synthase kinase 3 beta; PI3K alpha, Phosphatidylinositol 3 kinase regulatory subunit, alpha; SHP-2, Protein tyrosine phosphatase, nonreceptor type 11.

The resulting phosphoproteomic data presents a wide-scale view of the temporal changes of tyrosine phosphorylation events following TCR stimulation and inhibition of ERK activation. In all, from the SILAC experiments, 322 non-redundant tyrosine phosphopeptides on 243 proteins were identified at 1% FDR, of which 243 showed statistically significant U0126-responsive changes (q value <0.02). Kyoto Encyclopedia of Genes and Genomes (KEGG) pathway analysis was performed on identified sites (Figure 5) [35], [36]. Profound changes in the pattern of TCR signaling was observed in U0126-treated Jurkat T cells relative to control cells, as demonstrated in SILAC heatmaps, with overall decreases in phosphorylation of TCR proximal signaling proteins, including CD3 ε, δ, γ, ζ, Lck, and ZAP-70, as well as the adaptor and downstream signaling proteins VAV1, IL-2-inducible T cell kinase (Itk), phospholipase C γ1 (PLCγ1), and NCK1 (Figure 6, Figure 7). Despite deficiencies in the lipid phosphatases PTEN and SHIP1 in the Jurkat cell line [37], [38], the quantitative perturbations of phosphopeptide abundance observed with inhibition of ERK feedback in this study are consistent with observations made in primary T cells by Stefanova et al. [4] and Poltorak et al. [12] and support the hypothesis that ERK is a positive feedback regulator in TCR proximal signaling.

### Canonical TCR Signaling Proteins Exhibited Expected Phosphoproteomic Dynamics in Response to TCR Stimulation in DMSO-treated Control Cells

**Figure 8:**
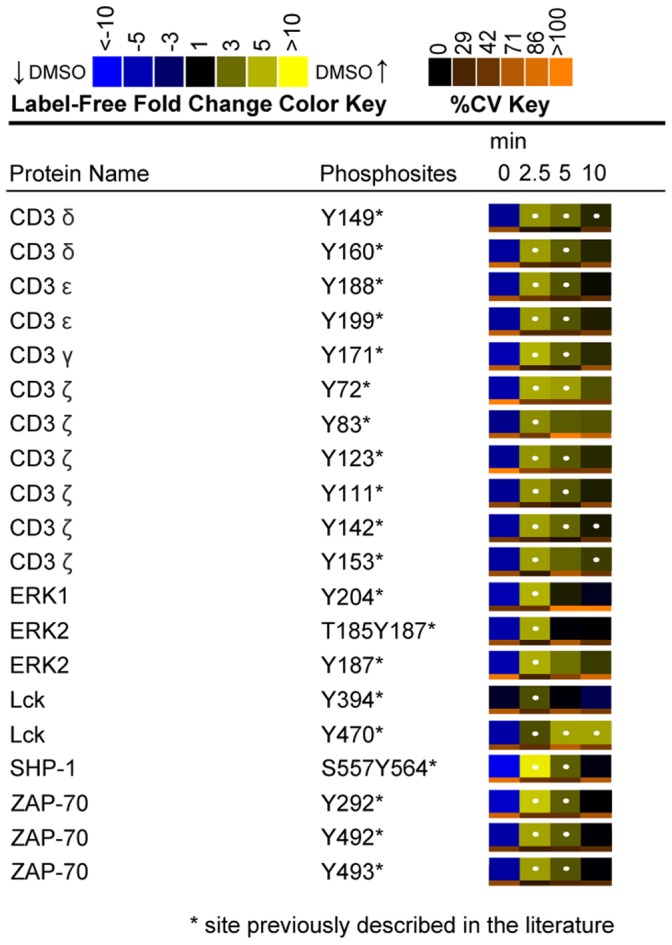
Label-free heatmaps of canonical TCR signaling proteins. Label-free heatmaps represent the temporal change in phosphorylation of DMSO-treated control Jurkat T cells through the time course of TCR stimulation. Heatmaps were calculated from the averages of four biological replicate experiments. In the label-free heatmaps, black color represents peptide abundance equal to the geometric mean for that peptide across all time points. Blue color represents peptide abundance less than the mean, whereas yellow corresponds to peptide abundance more than the mean. A white dot within a label-free heatmap square indicates a statistically significant difference (q value <0.02) in the fold change in peptide abundance for that time point in DMSO-treated control cells.

**Figure 9:**
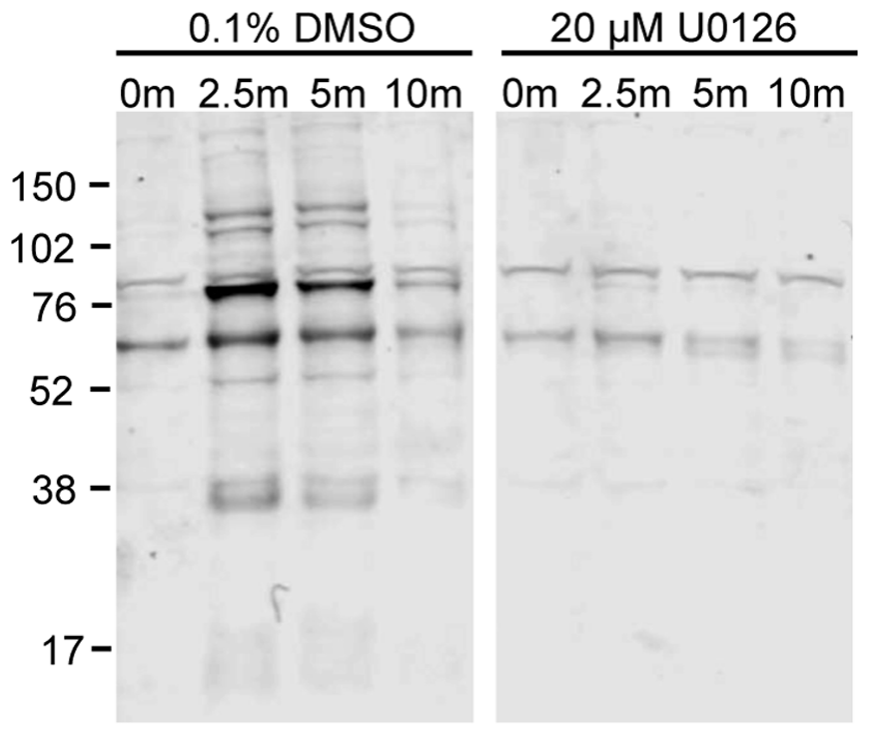
Tyrosine phosphorylation in U0126-treated and DMSO-treated Jurkat T cells across a time course of TCR stimulation. Cell lysates from one replicate of a time course of TCR stimulation in the presence of 0.1% DMSO or 20 µM U0126 were separated by SDS-PAGE and immunodetected with a monoclonal 4G10 antibody that recognizes phosphotyrosines. The immunoblot is representative of data from 4 biological replicate experiments.

Label-free heatmaps reflected the change in abundance of each phosphopeptide in DMSO-treated control Jurkat T cells across the four time points of TCR stimulation. Overall, label-free heatmap data followed expected patterns of TCR signaling phosphorylation dynamics, confirming T cell activation (Figure 8). In particular, statistically significant (q value <0.02) elevated phosphorylation of the CD3 ITAMs was observed, peaking at 2.5 minutes of TCR stimulation with an increase of greater than 14-fold from the 0 minute peak area. These observations are consistent with reports of ITAM phosphorylation kinetics at early TCR stimulation time points [16], [18]. Specific tyrosine residues on upstream TCR signaling proteins, including ZAP-70 (Tyr^492^, Tyr^493^, Tyr^292^), Lck (Tyr^394^, Tyr^470^), and SHP-1 (Tyr^564^) also showed statistically significant (q value <0.02) elevated phosphorylation with similar kinetics at 2.5 and 5 minutes of TCR stimulation. In addition, tyrosine sites on the downstream proteins ERK1 (Tyr^204^) and ERK2 (Tyr^187^) exhibited statistically significant (q value <0.02) elevated phosphorylation at the 2.5 minute time point, with average fold changes of 24.8 and 22.6 from the 0 minute peak areas respectively. These observations are consistent with observations made by other groups on the phosphorylation dynamics of these particular sites during TCR stimulation [16], [18], [39], [40]. In addition to the label-free heatmap data, 4G10 blots were performed to establish induction of signaling in response to TCR stimulation in DMSO-treated controls, as well as to confirm global decreases in signaling in response to inhibition of ERK feedback (Figure 9).

### The Majority of Canonical TCR Signaling Proteins Exhibited Decreased Tyrosine Phosphorylation in Response to Inhibition of ERK Activation

Of the confidently identified phosphopeptides in the analysis, 54 tyrosine phosphorylation sites on 27 proteins were found within the KEGG TCR signaling pathway, of which 42 phosphorylation sites on 20 proteins showed a statistically significant (q value <0.02) change in relative abundance upon inhibition of ERK activation (Figure 6, Figure 7). As expected, constitutive decreases in phosphorylation on the CD3 ζ ITAMs and Lck were observed, consistent with the original findings made by Stefanova et al. that illuminated ERK’s role in positive feedback regulation of TCR signaling [4]. The resulting phosphoproteomic SILAC data also revealed previously unobserved effects of ERK feedback perturbation on TCR signaling. Among these novel observations included statistically significant constitutive decreases in tyrosine phosphorylation of ZAP-70, Itk, PLCγ1, NCK1, and CD28.

U0126 treatment of Jurkat T cells had dramatic effects on the tyrosine phosphorylation dynamics of the Src kinase Lck across the time course of TCR stimulation. As a result of a miscleavage during tryptic digestion, a unique phosphopeptide containing Tyr^394^ (LIEDNEYpTAREGAK), the activation site of the Src family kinase Lck, was identified. This residue, a conserved tyrosine in the activation loop of Lck, exhibited a greater than 3-fold statistically significant (q value <0.0005) decrease in phosphorylation across all time points of TCR stimulation in U0126-treated cells relative to control cells. The phosphorylation of this site is a prerequisite for catalytic activity of Lck toward its cellular substrates [41]–[43]. Upon TCR engagement, this site is autophosphorylated, and dephosphorylation of this site is regulated primarily by SHP-1, as well as protein tyrosine phosphatase, nonreceptor, type 22 [44], [45]. This observation corroborates the model of competing ERK positive feedback and SHP-1 negative feedback pathways proposed by Stefanova et al. [4]. According to this model, the absence of ERK modification of Lck would allow SHP-1 binding to Lck and subsequent SHP-1-mediated inactivation of Lck by dephosphorylation at Tyr^394^ [4], [44].

Also observed on Lck was a greater than 3-fold statistically significant (q value <0.006) decrease in phosphorylation of Tyr^192^ across all time points in U0126-treated Jurkat cells relative to control cells. Tyr^192^ is a highly conserved site found in the SH2 domain of Lck that is phosphorylated in triggered T cells [46]. Phosphorylation of this site has been reported to induce a decrease in ligand binding by the SH2 domain of Lck, which in turn leads to activation of the kinase [46]. Given that ERK inhibition should allow greater access of SHP-1 to Lck, which is supported with the observation of decreased levels of Tyr^394^ phosphorylation on Lck, perhaps SHP-1 also acts on Tyr^192^. Follow-up biochemical experiments are necessary to further investigate this finding.

No unique phosphopeptide corresponding to the activation site of Fyn was identified, however, a number of putative substrates of Fyn kinase activity exhibited statistically significant (q value <0.02) decreases in phosphorylation in U0126-treated cells relative to control cells. These included Tyr^271^ of the adaptor protein SKAP55 and Tyr^571^ of the adhesion and degranulation promoting adaptor protein (ADAP), sites that are phosphorylated by Fyn in TCR-stimulated Jurkat T cells [47], [48]. Furthermore, Tyr^291^ of the hematopoietic-specific actin regulator Wiskott-Aldrich syndrome protein (WASP) showed a statistically significant (q value <0.016) decrease in phosphorylation in response to inhibition of ERK activation, a site phosphorylated by Fyn in TCR-stimulated cells [49]. Taken together, the data suggests that in addition to Lck, the Src kinase Fyn also exhibited decreased phosphorylation on its active site in response to U0126 treatment. Whether ERK directly or indirectly modifies the activity of Fyn is currently unknown. One hypothesis is that similar to ERK feedback on Ser^59^ Lck, ERK also directly modifies Fyn to increase its kinase activity. No amino acid sequence homologous to that surrounding Ser^59^ on Lck exist on Fyn, and it has been reported that the first 66 amino acids of Lck are unrelated to similarly placed unique regions in other Src kinases [15]. However, analysis with Scansite [50] identified Ser^307^ as a site that could be phosphorylated by ERK1. Further experimental work is needed to elucidate the regulation of Fyn by ERK.

Inhibition of the ERK feedback pathway not only resulted in decreases in phosphorylation on the active site of Lck, but also on a number of well-characterized Lck substrates. Tyrosine residues on the TCR ITAMs exhibited statistically significant (q value <0.02) decreases in phosphorylation across all time points following TCR stimulation of U0126-treated Jurkat T cells relative to control cells. Phosphorylation of ITAM domains on the ε, δ, γ, and ζ TCR subunits by Lck is key to the initiation of signaling cascades that characterize T cell activation [51]–[56]. These observations are in agreement with previous reports of inhibition of ERK activation in TCR engaged cells, where overall phosphorylation of the TCR CD3 ζ was decreased [4]. Another direct substrate of Lck, the Tec kinase Itk showed a greater than 2.7-fold statistically significant (q value <0.008) decrease in phosphorylation at Tyr^512^ at 0, 2.5, and 5 minutes of TCR stimulation in U0126-treated cells relative to controls. A conserved tyrosine residue in the activation loop, this site is reported to be phosphorylated by Lck in TCR cross-linked Jurkat cells to enhance Itk kinase activity [57]. In addition to the observed decreased phosphorylation of the active site of Itk, evidence for the attenuation of Itk kinase activity is supported by the identification of a greater than 1.6-fold statistically significant (q value <0.016) decrease in phosphorylation of Tyr^191^, Tyr^206^, and Tyr^209^ on CD28 in U0126-treated cells relative to control cells, sites shown to be phosphorylated by Itk [58].

**Figure 10:**
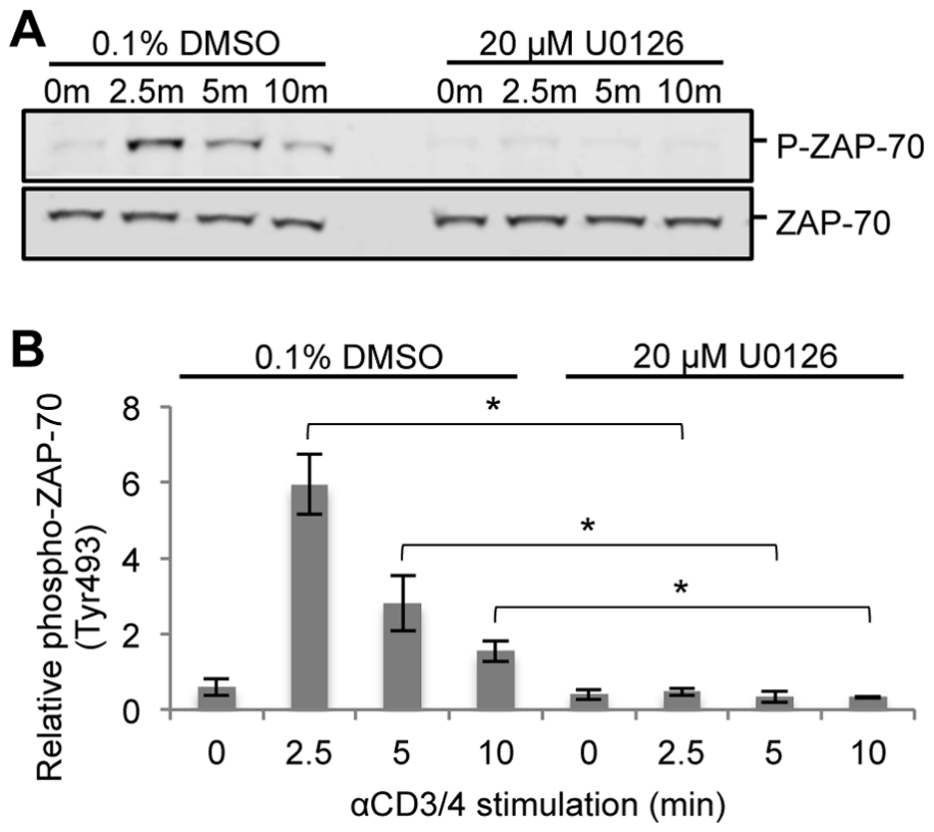
Phosphorylation of the activation site of ZAP-70 across a time course of TCR stimulation. (A) Cell lysates from one replicate of a time course of TCR stimulation in the presence of 0.1% DMSO or 20 µM U0126 were separated by SDS-PAGE and immunodetected with phospho-ZAP-70 (Tyr^493^) and ZAP-70 specific antibodies. (B) Densitometric analysis of phospho-ZAP-70 levels normalized to ZAP-70 levels was performed. Shown is the mean ± S.D. from 4 biological replicate experiments. Statistically significant differences in relative phospho-ZAP-70 levels between U0126-treated and DMSO-treated cells for each time point are indicated with an asterisk (*-p value <0.003).

Another consequence of U0126-mediated inhibition of ERK activation was constitutive, statistically significant (q value <0.02) decreases in phosphorylation of Tyr^69^, Tyr^292^, Tyr^397^, Tyr^492^, and Tyr^493^ on ZAP-70. It is well established that Lck mediates the phosphorylation of ZAP-70 at Tyr^493^ to increase its kinase activity in stimulated T cells [59]. A greater than 6-fold statistically significant (q value <0.0007) decrease in phosphorylation of the activation site of ZAP-70 at the 2.5, 5 and 10 minute time points of TCR stimulation was observed in U0126-treated cells relative to controls. This observation recapitulates the ERK positive feedback model as inhibition of ERK activation led to decreased Lck phosphorylation at its activation site, as well as decreased phosphorylation of its substrate, ZAP-70. Western blot analysis of phospho-ZAP-70 (Tyr^493^) across all 4 time points of TCR stimulation demonstrated induction of phosphorylation of the site in control cells and recapitulated the MS observations of decreased phosphorylation of the site in U0126-treated cells (Figure 10A, B). Both Tyr^492^ and Tyr^292^ autophosphorylation on ZAP-70 is dependent on the initial phosphorylation of Tyr^493^ [60]. The observed greater than 5-fold statistically significant (q value <0.007) decreases across the majority of time points on these two sites are thus likely a consequence of decreased Lck and ZAP-70 catalytic activity.

Although the majority of ZAP-70 tyrosine sites displayed decreased phosphorylation in response to inhibition of ERK feedback, one particular residue on the C-terminus of the protein, Tyr^598^, showed a greater than 1.7-fold statistically significant (q value <0.009) constitutive increase in phosphorylation at 0 and 10 minutes of TCR stimulation. The importance of this residue has been highlighted by a previous study that reported a gain-of-function phenotype in Jurkat T cells when the residue was mutated to phenylalanine [61]. Furthermore, analysis of the crystal structure of ZAP-70 highlighted the importance of Tyr^598^ in maintaining a ZAP-70 autoinhibitory state in resting T cells through stabilization of the “linker-kinase sandwich,” the defining aspect of the inactive ZAP-70 structure [61]. It has been suggested that binding of ZAP-70 to phosphorylated ITAM motifs promotes disassembly of the “linker-kinase sandwich,” which is further promoted by phosphorylation of Tyr^598^ and other tyrosine residues at the activation loop of the kinase [62]. No kinases or phosphatases have been identified that directly act on this particular tyrosine residue. However, the observed decreases in phosphorylation on the majority of protein tyrosine kinases proximal to the TCR in response to U0126 treatment suggest that the elevated phosphorylation of this site is unlikely a consequence of kinase activity.

### Tyrosine Phosphorylation of Proteins Found within the KEGG Regulation of Actin Cytoskeleton Pathway also Exhibited Overall Decreased Phosphorylation in Response to Inhibition of ERK Activation

**Figure 11:**
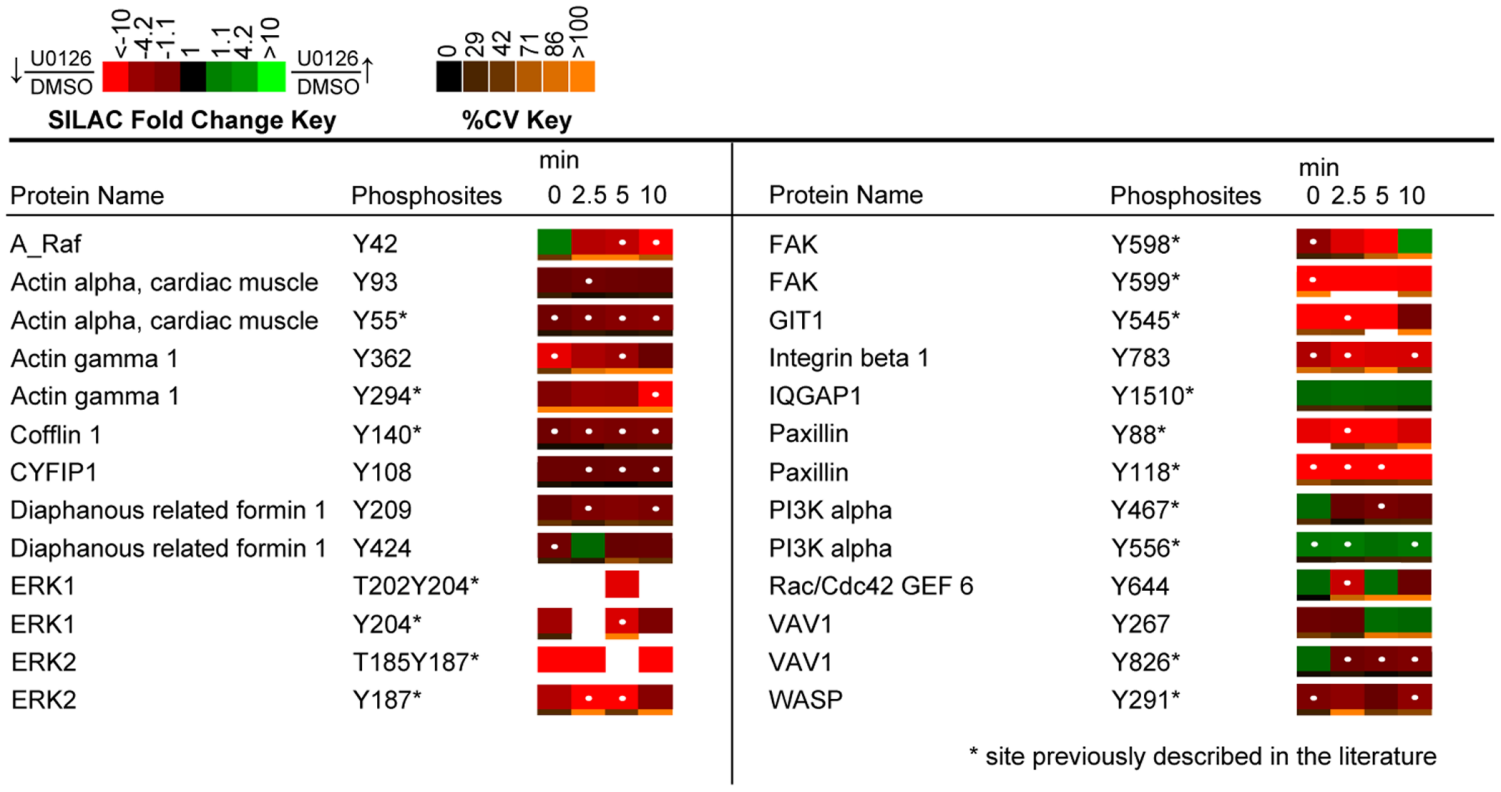
Quantitative phosphoproteomic analysis of proteins associated with the KEGG regulation of actin cytoskeleton category. Heatmaps were calculated from the averages of four biological replicate experiments. White dots within the SILAC heatmaps indicate a statistically significant difference (q value <0.02) in the comparison between U0126-treated and DMSO-treated control Jurkat T cell SILAC ratios for that time point. Abbreviations: A_Raf: A-Raf proto-oncogene; CYFIP1, Cytoplasmic FMR1 interacting protein 1; GIT1, G protein-coupled receptor kinase-interactor 1; IQGAP1, IQ motif containing GTPase activating protein 1.

The results of this phosphoproteomic investigation also suggest interplay between ERK positive feedback signaling and proteins involved in the regulation of actin cytoskeleton pathway. A total of 24 tyrosine phosphorylation sites on 17 proteins identified at high confidence were from proteins within the KEGG regulation of actin cytoskeleton pathway (Figure 11). Of these identified phosphopeptides, 22 sites on 16 proteins showed a statistically significant (q value <0.02) change in relative abundance between U0126-treated and DMSO-treated control cells. Again, constitutive decreases in phosphorylation were found on the majority of tyrosine sites that showed statistically significant changes in relative abundance.

Actin filaments play a fundamental role in antigen recognition through regulation of the formation of an immunological synapse, and the formation of scaffolds for various signaling complexes [63]. Various studies have illuminated the requirement of TCR-proximal signaling molecules for actin accumulation at the immunological synapse, including Lck, ZAP-70, and Itk [64]–[66]. Observed U0126-mediated decreases in tyrosine phosphorylation on upstream TCR signaling proteins would thus be expected to attenuate signaling of actin regulatory proteins. The phosphoproteomic dataset generated in this study is the first to demonstrate global decreases in phosphorylation of proteins essential to actin regulation with inhibition of ERK feedback in Jurkat T cells. Amongst the phosphorylation sites of important actin-regulatory proteins quantified included Tyr^826^ of VAV1, a site implicated in VAV1 GEF activity that showed a statistically significant (q value <0.016) decrease in phosphorylation at 2.5, 5 and 10 minutes of TCR stimulation in response to U0126 treatment [67]. Furthermore, as previously mentioned, Tyr^291^ of WASP showed a constitutive decrease in phosphorylation, with statistically significant (q value <0.016) changes at 0 and 10 minutes of TCR stimulation. Mutation of this site has been shown to abrogate WASP effector functions, including actin polymerization and immunological synapse formation [49].

### Tyrosine Phosphorylation of Proteins Implicated in Integrin Signaling also Exhibited Overall Decreased Phosphorylation in Response to Inhibition of ERK Activation

**Figure 12:**
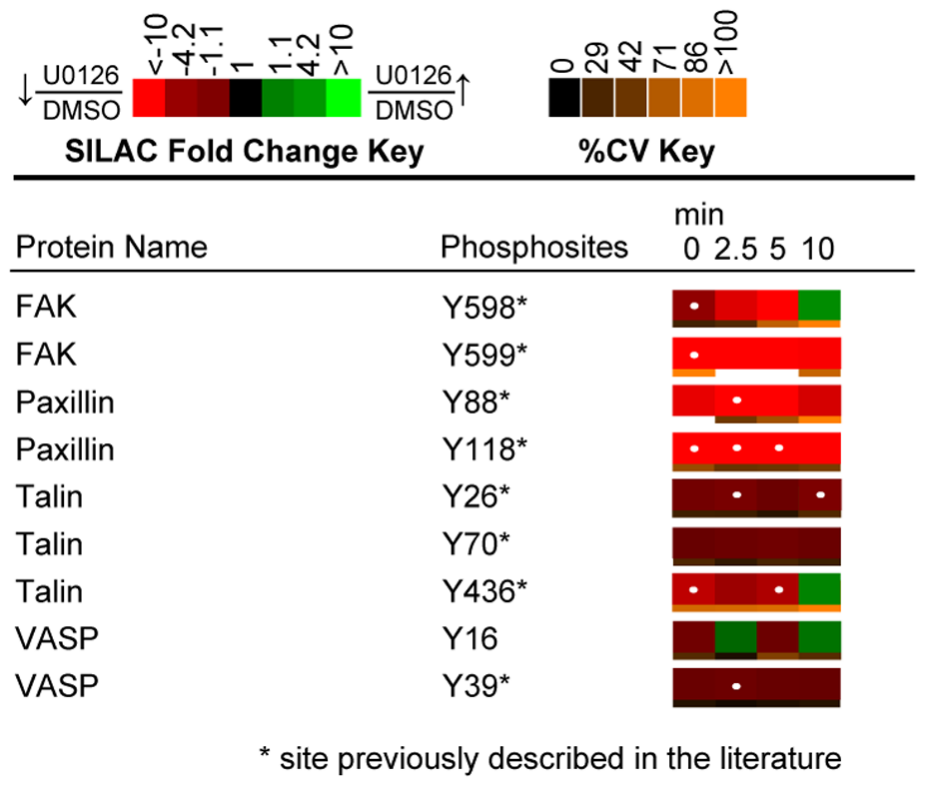
Quantitative phosphoproteomic analysis of proteins implicated in integrin signaling. Heatmaps were calculated from the averages of four biological replicate experiments. White dots within the SILAC heatmaps indicate a statistically significant difference (q value <0.02) in the comparison between U0126-treated and DMSO-treated control Jurkat T cell SILAC ratios for that time point.

KEGG analysis identified a number of proteins implicated in the focal adhesion pathway. Structures that resemble focal adhesions have not been identified in T cells; however, a similar organization of signaling molecules may occur in TCR “inside out” signaling, which is essential to integrin activation. Proteins identified in the dataset that are implicated in “inside out” signaling included vasodilator-stimulated phosphoprotein (VASP), focal adhesion kinase (FAK), talin, and paxillin (Figure 12) [68]. Phosphorylation of Tyr^598^ of FAK by Src family kinases is essential to the activation of the protein tyrosine kinase [69]. U0126 treatment of Jurkat cells resulted in a statistically significant (q value <0.02) decrease in the phosphorylation of this site relative to controls. Furthermore, a substrate of FAK kinase activity, Tyr^118^ on paxillin also showed a greater than 7-fold statistically significant (q value <0.012) decrease in phosphorylation at 0, 2.5, and 5 minutes of TCR stimulation in U0126-treated cells relative to control cells [70]. These findings suggest that ERK feedback may also affect pathways leading to the activation of integrin signaling in Jurkat T cells.

### Conclusions

A wide-scale quantitative phosphoproteomic analysis of U0126-treated Jurkat T cells has been performed with the objective of elucidating the phosphorylation events that characterize ERK feedback in TCR signaling. Using a combination of label-free and SILAC quantification techniques, subtle fluctuations of cellular tyrosine signaling networks in response to ERK perturbation were captured and quantified over time. From this analysis, new insights into the critical role of ERK feedback in the regulation of the dynamic tyrosine phosphoproteome after TCR engagement in Jurkat T cells have been provided. Future studies will apply the unbiased and wide-scale approach demonstrated here to primary T cells with the hopes of better understanding the role of ERK positive feedback in TCR signaling.

## Supporting Information

Figure S1 **Titration to determine optimal conditions for U0126 inhibition of MEK1/2.** Jurkat T cells were treated with various concentrations (0 µM, 10 µM, 20 µM) of the MEK1/2 inhibitor for multiple incubation periods (0, 2, 2.5 hours). 0 µM samples were treated with 0.1% DMSO, as this was the background control. Inhibition was determined using immunoblots. After U0126 treatment and TCR stimulation, cell lysates were separated by SDS-PAGE and immunoblotted with a phospho-ERK1/2 specific antibody. Densitometric analysis was performed on relative levels of phospho-ERK1/2. Shown is the mean ± S.D. from 3 biological replicate experiments.
(TIF)

Figure S2 **Quantification of ERK levels after inhibitor treatment.** Jurkat T cells incubated with either 0.1% DMSO or 20 µM U0126 for 2.5 hours were separated by SDS-PAGE and immunobloted with antibodies against ERK1/2 and GAPDH. Densitometric analysis was performed on relative levels of ERK1/2. Shown is the mean ± S.D. from 4 biological replicate experiments.
(TIF)

Figure S3 **Assessment of the reproducibility of SILAC ratios amongst the four biological replicate experiments.** Scatter plots of SILAC ratios (log2 transformed) from four replicate experiments demonstrated good correlation and thus reproducibility.
(TIF)

Dataset S1 **Quantitative and statistic analysis of all identified phosphopeptides.** Sequence and phosphorylation site assignment of all identified phosphopeptides with their corresponding SIC peak areas and statistics (CV and q values) from both U0126-treated and DMSO-treated control Jurkat T cells.
(XLS)
