# Supplementary figures and images for: ERK Positive Feedback Regulates a Widespread Network of Tyrosine Phosphorylation Sites across Canonical T Cell Signaling and Actin Cytoskeletal Proteins in Jurkat T Cells
Source: PLoS One. 2013 Jul 17;8(7):e69641. doi: 10.1371/journal.pone.0069641 (PMC3714263; doi:10.1371/journal.pone.0069641)

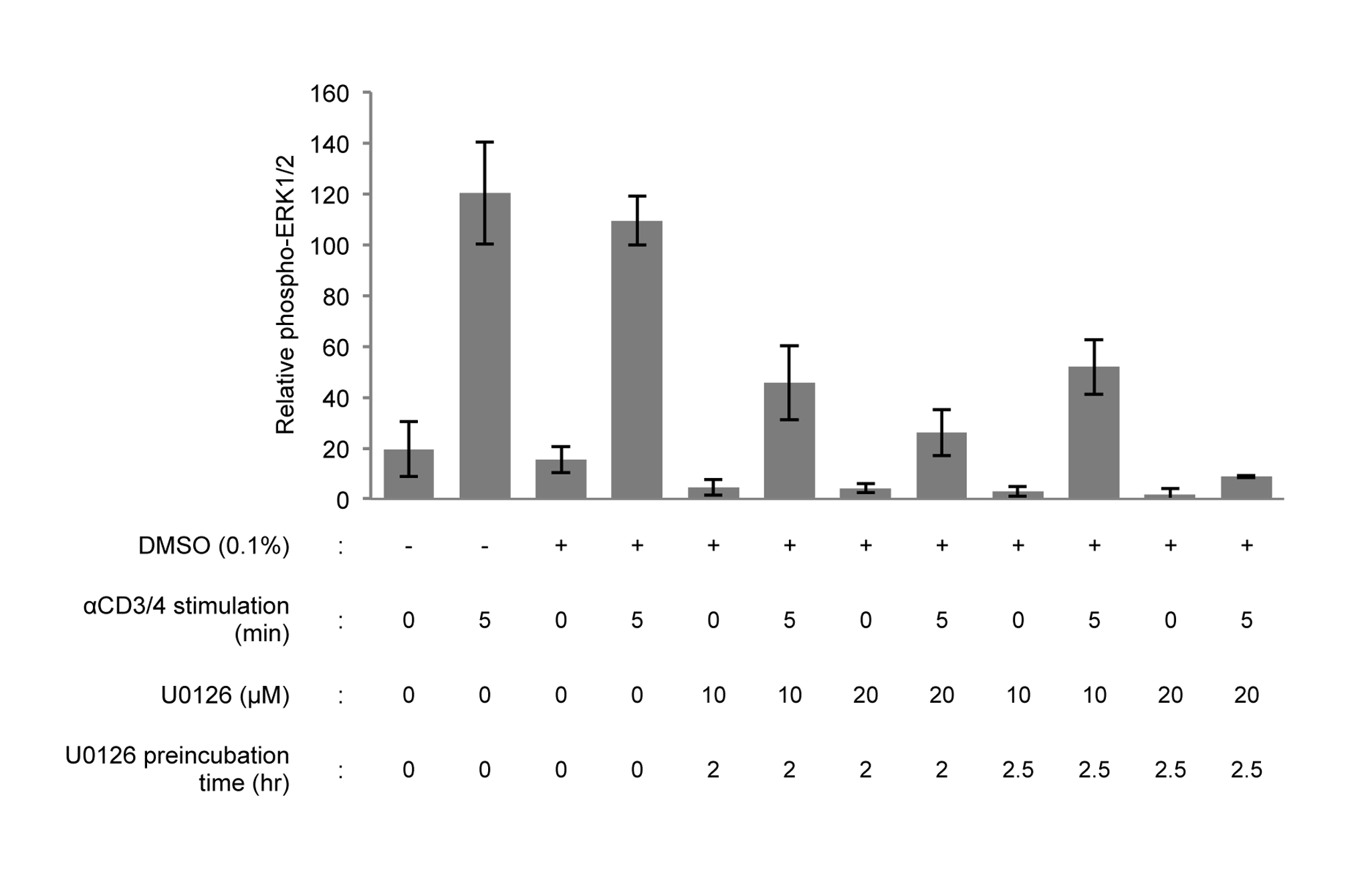

Supplement: Figure S1 — Titration to determine optimal conditions for U0126 inhibition of MEK1/2. Jurkat T cells were treated with various concentrations (0 µM, 10 µM, 20 µM) of the MEK1/2 inhibitor for multiple incubation periods (0, 2, 2.5 hours). 0 µM samples were treated with 0.1% DMSO, as this was the background control. Inhibition was determined using immunoblots. After U0126 treatment and TCR stimulation, cell lysates were separated by SDS-PAGE and immunoblotted with a phospho-ERK1/2 specific antibody. Densitometric analysis was performed on relative levels of phospho-ERK1/2. Shown is the mean ± S.D. from 3 biological replicate experiments. (TIF) [file pone.0069641.s001.tif]

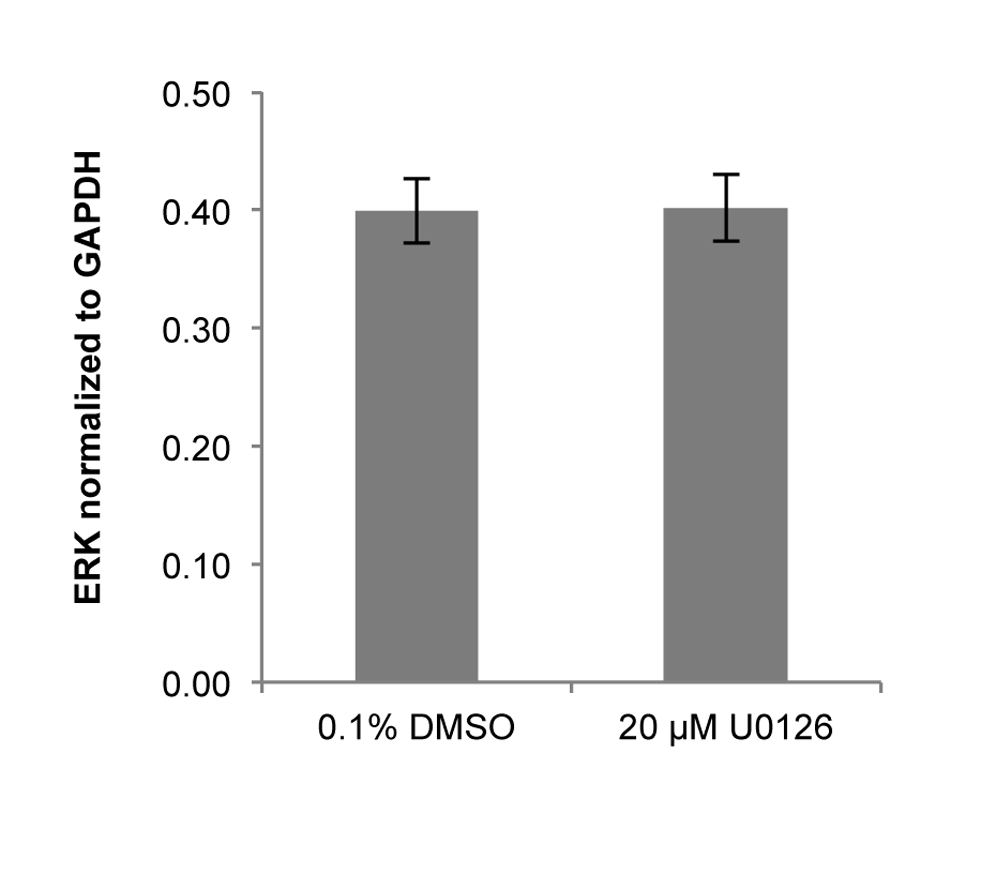

Supplement: Figure S2 — Quantification of ERK levels after inhibitor treatment. Jurkat T cells incubated with either 0.1% DMSO or 20 µM U0126 for 2.5 hours were separated by SDS-PAGE and immunobloted with antibodies against ERK1/2 and GAPDH. Densitometric analysis was performed on relative levels of ERK1/2. Shown is the mean ± S.D. from 4 biological replicate experiments. (TIF) [file pone.0069641.s002.tif]

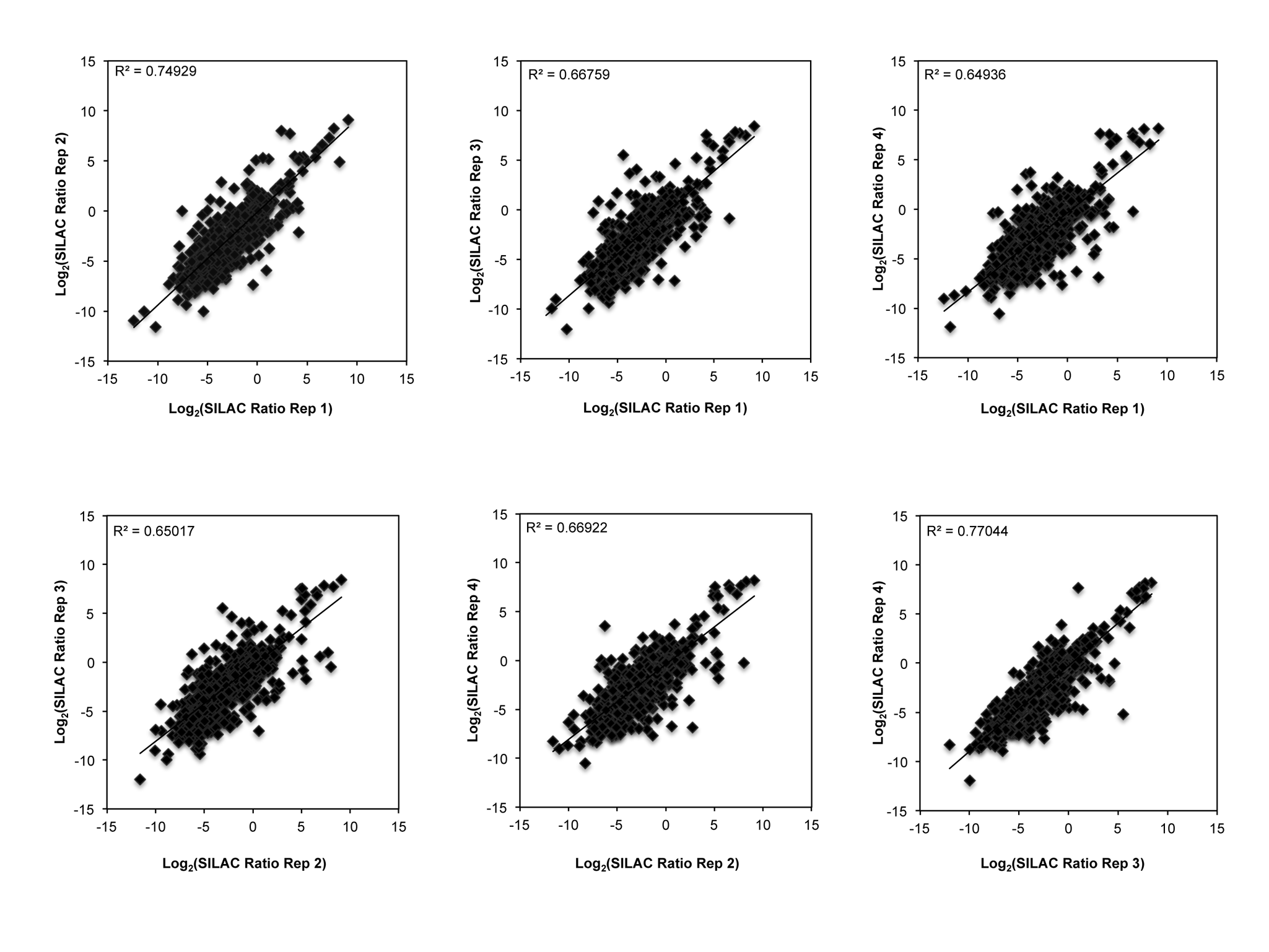

Supplement: Figure S3 — Assessment of the reproducibility of SILAC ratios amongst the four biological replicate experiments. Scatter plots of SILAC ratios (log2 transformed) from four replicate experiments demonstrated good correlation and thus reproducibility. (TIF) [file pone.0069641.s003.tif]
